# Supplementary material for: Validating methods for inferring co-occurring diseases: a flexible framework for simulating synthetic data
Source: BMC Med Res Methodol. 2026 Aug 19;26:184. doi: 10.1186/s12874-026-02966-2 (PMC13488252; doi:10.1186/s12874-026-02966-2)
Supplement: Supplementary file 1 [file 12874_2026_2966_MOESM1_ESM.pdf]

Appendix: Validating methods for inferring co-occurring diseases: a flexible framework for simulating synthetic data

A Supplementary Material

A.1 Derivation of log-odds for disease combinations in multinomial model

In the following, we consider the log-odds of the joint occurrence of diseases, based on information about the exclusive occurrence of single diseases. The derived formulas are employed in Section 2.2.2, Approach IId, Step 1, Option (iii). We focus on the joint consideration of two and three different but potentially related diseases. The derived relationships can also be extended to more than three diseases, and the formulas can be employed as a sub-model in the context of $d > 3$ co-occurring diseases.

To start with, we look at the scenario of two diseases A and B. Let Y be a multinomial random variable denoting the disease status of a particular patient which can take the values A (meaning that the patient suffers exclusively from disease A), B (analogously), AB (both diseases occur simultaneously) of H (the patient is healthy, the reference category). By π_A, π_B, π_{AB} and π_H we denote the probability for Y taking the respective value. Additionally, we introduce binary random variables $\underline{A}$ and $\underline{B}$ indicating whether the patient suffers from the respective disease (value one) or not.

The log-odds for Y taking the values A, B and AB are defined as

$$\eta_A = \log\left(\frac{\pi_A}{\pi_H}\right) = \log\left(\frac{P(Y=A)}{P(Y=H)}\right), \tag{1}$$

$$\eta_B = \log\left(\frac{\pi_B}{\pi_H}\right) = \log\left(\frac{P(Y=B)}{P(Y=H)}\right) \tag{2}$$

and

$$\eta_{AB} = \log\left(\frac{\pi_{AB}}{\pi_H}\right) = \log\left(\frac{P(Y=AB)}{P(Y=H)}\right) \tag{3}$$

respectively. The odds ratio (OR) for a 2×2 contingency table is often used as a measure of association [1]. We will make use of it to determine the desired association between two diseases. We first calculate the log-odds ratio for diseases A and B, using the definition for OR from [2], as

$$\begin{aligned}
\log(OR_{\underline{A},\underline{B}}) &= \log\left(\frac{P(\underline{A}=1,\underline{B}=1)}{P(\underline{A}=1,\underline{B}=0)} \Big/ \frac{P(\underline{A}=0,\underline{B}=1)}{P(\underline{A}=0,\underline{B}=0)}\right) \\
&= \log\left(\frac{P(\underline{A}=1,\underline{B}=1)\cdot P(\underline{A}=0,\underline{B}=0)}{P(\underline{A}=1,\underline{B}=0)\cdot P(\underline{A}=0,\underline{B}=1)}\right) \\
&= \log\left(\frac{P(Y=AB)\cdot P(Y=H)}{P(Y=A)\cdot P(Y=B)}\right) \\
&= \log(P(Y=AB)) + \log(P(Y=H)) - \log(P(Y=A)) - \log(P(Y=B)).
\end{aligned} \tag{4}$$

Equations (1), (2) and (3) can be rearranged to the following:

$$\begin{aligned}
\log(P(Y=A)) &= \eta_A + \log(P(Y=H)), \\
\log(P(Y=B)) &= \eta_B + \log(P(Y=H)), \\
\log(P(Y=AB)) &= \eta_{AB} + \log(P(Y=H)).
\end{aligned} \tag{5}$$

Plugging in the formulas from Equation (5) into Equation (4), we can write

$$\begin{aligned}
&\log(OR_{\underline{A},\underline{B}}) \\
&= \eta_{AB} + \log(P(Y=H)) + \log(P(Y=H)) - \eta_A - \log(P(Y=H)) - \eta_B - \log(P(Y=H)) \\
&= \eta_{AB} - \eta_A - \eta_B.
\end{aligned}$$

Thus, we can calculate the linear predictor for category AB as

$$\eta_{AB} = \log(OR_{\underline{A},\underline{B}}) + \eta_A + \eta_B,$$

where we set $OR_{\underline{A},\underline{B}}$ to the desired association between diseases A and B.

For three diseases A, B and C (and respective definitions of random variables as above), the linear predictor η_{ABC} for disease combination ABC can be calculated as described in the following. The possible disease occurrence combinations are A, B, C, AB, AC, BC, ABC and H. Additionally

to Equations (1)-(3), we define the following:

$$\eta_C = \log\left(\frac{\pi_C}{\pi_H}\right) = \log\left(\frac{P(Y=C)}{P(Y=H)}\right) \tag{6}$$

and

$$\eta_{ABC} = \log\left(\frac{\pi_{ABC}}{\pi_H}\right) = \log\left(\frac{P(Y=ABC)}{P(Y=H)}\right). \tag{7}$$

Analogously to Equation (5), we can rearrange η_C and η_{ABC} accordingly:

$$\begin{aligned} \log(P(Y=C)) &= \eta_C + \log(P(Y=H)), \\ \log(P(Y=ABC)) &= \eta_{ABC} + \log(P(Y=H)). \end{aligned} \tag{8}$$

To calculate η_{ABC} for disease combination ABC, we use the log-odds ratio for diseases A, B and C, which can be defined by the ratio of the two conditional log-odds ratios:

$$\log(OR_{\underline{A},\underline{B},\underline{C}}) = \frac{\log(OR_{\underline{A},\underline{B}|\underline{C}=1})}{\log(OR_{\underline{A},\underline{B}|\underline{C}=0})} \tag{9}$$

The conditional log-odds ratios can be seen as two different 2×2 contingency tables and therefore, similar to Equation (4), calculated as shown here:

$$\begin{aligned} \log(OR_{\underline{A},\underline{B}|\underline{C}=1}) &= \log\left(\frac{P(\underline{A}=1,\underline{B}=1,\underline{C}=1)}{P(\underline{A}=1,\underline{B}=0,\underline{C}=1)} \Big/ \frac{P(\underline{A}=0,\underline{B}=1,\underline{C}=1)}{P(\underline{A}=0,\underline{B}=0,\underline{C}=1)}\right) \\ &= \log\left(\frac{P(\underline{A}=1,\underline{B}=1,\underline{C}=1)\cdot P(\underline{A}=0,\underline{B}=0,\underline{C}=1)}{P(\underline{A}=1,\underline{B}=0,\underline{C}=1)\cdot P(\underline{A}=0,\underline{B}=1,\underline{C}=1)}\right) \\ &= \log\left(\frac{P(Y=ABC)\cdot P(Y=C)}{P(Y=AC)\cdot P(Y=BC)}\right) \\ &= \log(P(Y=ABC)) + \log(P(Y=C)) - \log(P(Y=AC)) - \log(P(Y=BC)) \\ &= \eta_{ABC} + \eta_C - \eta_{AC} - \eta_{BC} \end{aligned} \tag{10}$$

and

$$
\begin{aligned}
\log(OR_{\underline{A},\underline{B}|\underline{C}=0}) &= \log\left(\frac{P(\underline{A}=1,\underline{B}=1,\underline{C}=0)}{P(\underline{A}=1,\underline{B}=0,\underline{C}=0)} \Bigg/ \frac{P(\underline{A}=0,\underline{B}=1,\underline{C}=0)}{P(\underline{A}=0,\underline{B}=0,\underline{C}=0)}\right) \\
&= \log\left(\frac{P(\underline{A}=1,\underline{B}=1,\underline{C}=0)\cdot P(\underline{A}=0,\underline{B}=0,\underline{C}=0)}{P(\underline{A}=1,\underline{B}=0,\underline{C}=0)\cdot P(\underline{A}=0,\underline{B}=1,\underline{C}=0)}\right) \\
&= \log\left(\frac{P(Y=AB)\cdot P(Y=H)}{P(Y=A)\cdot P(Y=B)}\right) \\
&= \log(P(Y=AB)) + \log(P(Y=H)) - \log(P(Y=A)) - \log(P(Y=B)) \\
&= \eta_{AB} - \eta_A - \eta_B.
\end{aligned}
\tag{11}
$$

Using Equations (10) and (11) in Equation (9), we get

$$
\begin{aligned}
\log(OR_{\underline{A},\underline{B},\underline{C}}) &= \log(OR_{\underline{A},\underline{B}|\underline{C}=1}) - \log(OR_{\underline{A},\underline{B}|\underline{C}=0}) \\
&= \eta_{ABC} + \eta_C - \eta_{AC} - \eta_{BC} - \eta_{AB} + \eta_A + \eta_B.
\end{aligned}
\tag{12}
$$

Finally, rearranging Equation (12), the linear predictor for disease combination ABC can be defined as:

$$
\eta_{ABC} = \log(OR_{\underline{A},\underline{B},\underline{C}}) - \eta_{AB} - \eta_{AC} - \eta_{BC} + \eta_A + \eta_B + \eta_C. \tag{13}
$$

A.2 Required input specifications for Step II

Table A1 summarizes required input specifications for the model-based Approaches IIa to IId and the correlation-based Approaches Ib and IVe.

Approach	Required parameter
**IIa**	- Vector of k covariates for patients $i = 1, \dots, n$: $\boldsymbol{x}_i = (x_{i1}, \dots, x_{ik})'$ (obtained by Step I)<br>- Vector of regression coefficients for each disease $j \in \{1, \dots, d\}$: $\boldsymbol{\beta}_j = (\beta_{j0}, \dots, \beta_{jk})'$
**IIb**	- Vector of k covariates for patients $i = 1, \dots, n$: $\boldsymbol{x}_i = (x_{i1}, \dots, x_{ik})'$ (obtained by Step I)<br>- Vector of regression coefficients for each disease $j \in \{1, \dots, d\}$: $\boldsymbol{\beta}_j = (\beta_{j0}, \dots, \beta_{jk})'$<br>- Coefficients γ_{jl} which describe how the occurrence probability of disease l is influenced by the occurrence of diseases $j \in \{1, \dots, l-1\}$
**IIc**	- Vector of k covariates for patients $i = 1, \dots, n$: $\boldsymbol{x}_i = (x_{i1}, \dots, x_{ik})'$ (obtained by Step I)<br>- Vector of regression coefficients for each disease $j \in \{1, \dots, d\}$: $\boldsymbol{\beta}_j = (\beta_{j0}, \dots, \beta_{jk})'$<br>- Matrix $\boldsymbol{B}^*$ defining which diseases j and l share a latent factor for $j, l \in \{1, \dots, d\}$<br>- Set of coefficients $\lambda_{jl} \in \mathbb{R}$ which indicate the influence of the latent factor shared by diseases j and l on disease j
**IId**	- Vector of k covariates for patients $i = 1, \dots, n$: $\boldsymbol{x}_i = (x_{i1}, \dots, x_{ik})'$ (obtained by Step I)<br>- Alternatives:<br>(i) Vector of regression coefficients for each disease combination $r \in \{2, \dots, 2^d\}$: $\boldsymbol{\beta}_r = (\beta_{r0}, \dots, \beta_{rk})'$<br>(ii) Vector of regression coefficients for each disease $j \in \{1, \dots, d\}$: $\boldsymbol{\beta}_j = (\beta_{j0}, \dots, \beta_{jk})'$<br>(iii) Vector of regression coefficients for each single disease $j \in \{1, \dots, d\}$: $\boldsymbol{\beta}_j = (\beta_{j0}, \dots, \beta_{jk})'$; additionally odds ratios OR_{ir} which quantify for patient i the association between the single diseases contained in combination r
**IVe**	- Vector of k covariates for patients $i = 1, \dots, n$: $\boldsymbol{x}_i = (x_{i1}, \dots, x_{ik})'$ (obtained by Step I)<br>- $(d \times d)$-dimensional matrix $\boldsymbol{R}_{yy}$ with pairwise correlations between all d diseases<br>- $(d \times k)$-dimensional matrix $\boldsymbol{R}_{yx}$ with pairwise correlations between all d diseases and k covariates<br>- Marginal cumulative distribution functions $F_{y,j}$ for $j \in \{1, \dots, d\}$<br>- Prevalence p_j for each individual disease j

Table A1: Required input specifications for Approaches IIa to IId and IVe. Here, we assume that Step I has already been completed by using either Approach Ia or Ib. In addition to the listed parameters, one needs to specify prevalences p_j for each individual disease j (for Approaches IIa to IIc) or p_r for each disease combination r (for Approach IId) to proceed to Steps III and IV.

A.3 Parameter values for use case

Tables A2 and A3 list parameters that were derived from real data (with standardized metric covariates) and used as input values for synthetic data generation in the use case in Section 2.4. These are correlation coefficients among and between covariates and disease occurrences for the correlation-based Approaches Ib (all paths) and IVe (Path E) in Table A2, and regression coefficients for the model-based Approaches IIa to IId (Paths A to D) in Table A3.

	IMID	FMS	Age	Sex	CRP	FiRST	NRS	Tender
IMID	1.00	-0.70	0.20	0.27	0.35	-0.18	0.05	-0.22
FMS	-0.70	1.00	-0.18	-0.52	-0.22	0.57	0.28	0.83
Age	0.20	-0.18	1.00	0.04	0.21	0.00	0.13	-0.02
Sex	0.27	-0.52	0.04	1.00	0.17	-0.25	-0.10	-0.43
CRP	0.35	-0.22	0.21	0.17	1.00	-0.06	0.13	-0.07
FiRST	-0.18	0.57	0.00	-0.25	-0.06	1.00	0.40	0.53
NRS	0.05	0.28	0.13	-0.10	0.13	0.40	1.00	0.33
Tender	-0.22	0.83	-0.02	-0.43	-0.07	0.53	0.33	1.00

Table A2: Input correlations for data generation derived from real data. Path E uses the full matrix; for Paths A to D, only rows/columns 3 to 8 are relevant. Correlation was computed as follows: For pairs of variables that are both continuous in the real data, we use Pearson's correlation coefficient [3]; for pairs of binary variables, we calculate the tetrachoric correlation (see Section 2.3); for pairs containing one continuous and one binary variable, we calculate the polyserial correlation [4, 5].

	β_{IMID}	β_{FMS}	β_{onlyIMID}	β_{onlyFMS}	$\beta_{\text{IMID\&FMS}}$
(Intercept)	0.01	-1.96	0.52	-0.44	-2.45
Age	0.17	-0.45	0.15	-0.34	-0.60
Sex	0.10	-0.87	-0.04	-1.03	-0.12
CRP	2.32	-2.04	2.18	-1.18	1.08
FiRST	-0.10	0.63	-0.02	0.62	0.67
NRS	0.14	0.21	0.12	0.19	0.85
Tender	-0.31	1.39	0.30	1.57	1.45

Table A3: Input regression coefficients for data generation in Step II of Paths A to D, derived from real data as described in Section 2.4.2.

A.4 Use case: Additional results

We provide additional results for the use case in Section 2.4: Just like Figure 2 in the main manuscript, Figures A1 to A5 display relative differences between the estimated value of the regression coefficient or correlation coefficient and the input value; here for all covariates other than `Age`. Table A4 lists estimated values of the regression coefficient or correlation coefficient, and Table A5 shows empirical disease prevalence and disease association in simulated data.

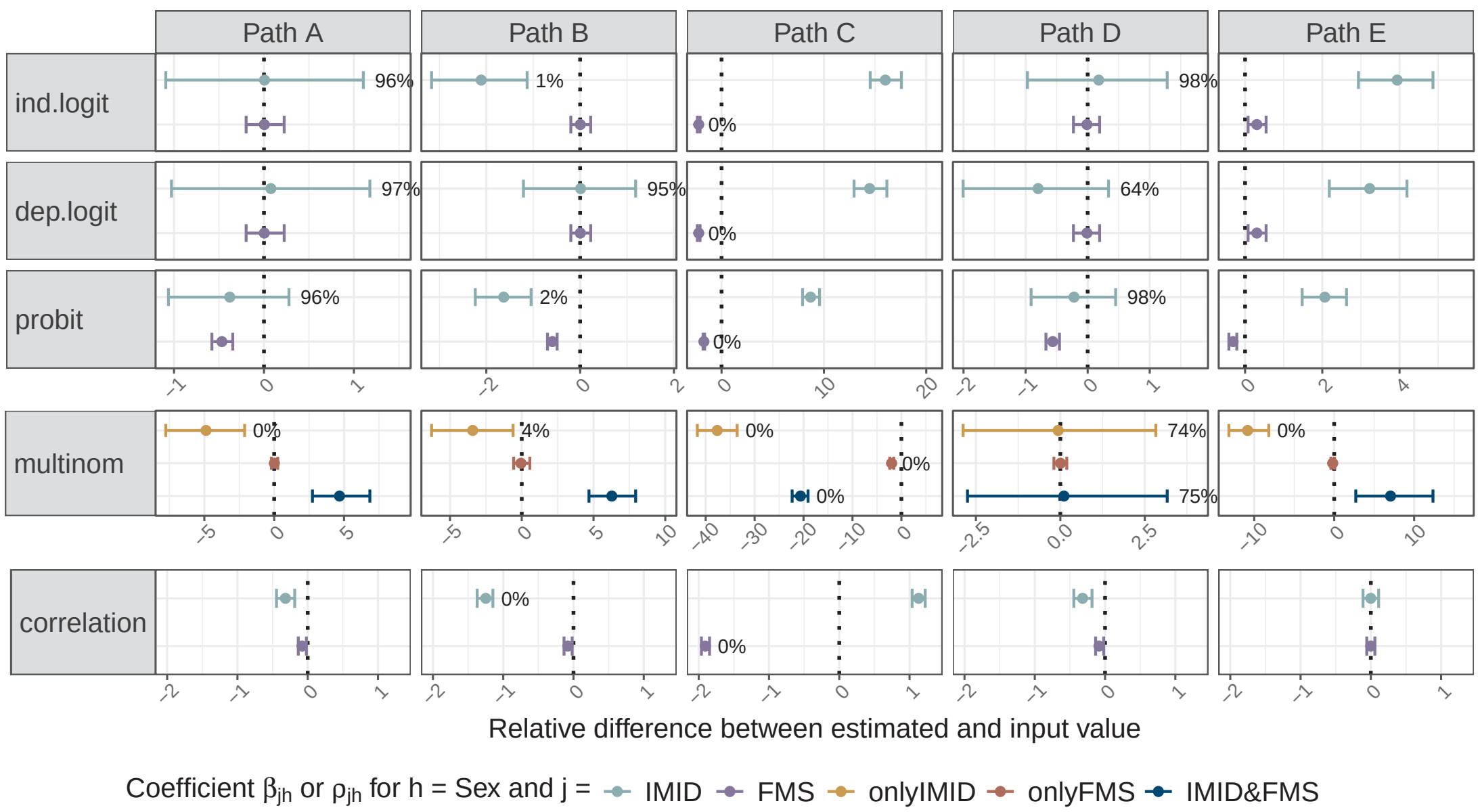

Figure A1: Simulation results as in Figure 2 in the main manuscript, this time for covariate $h =$ `Sex`.

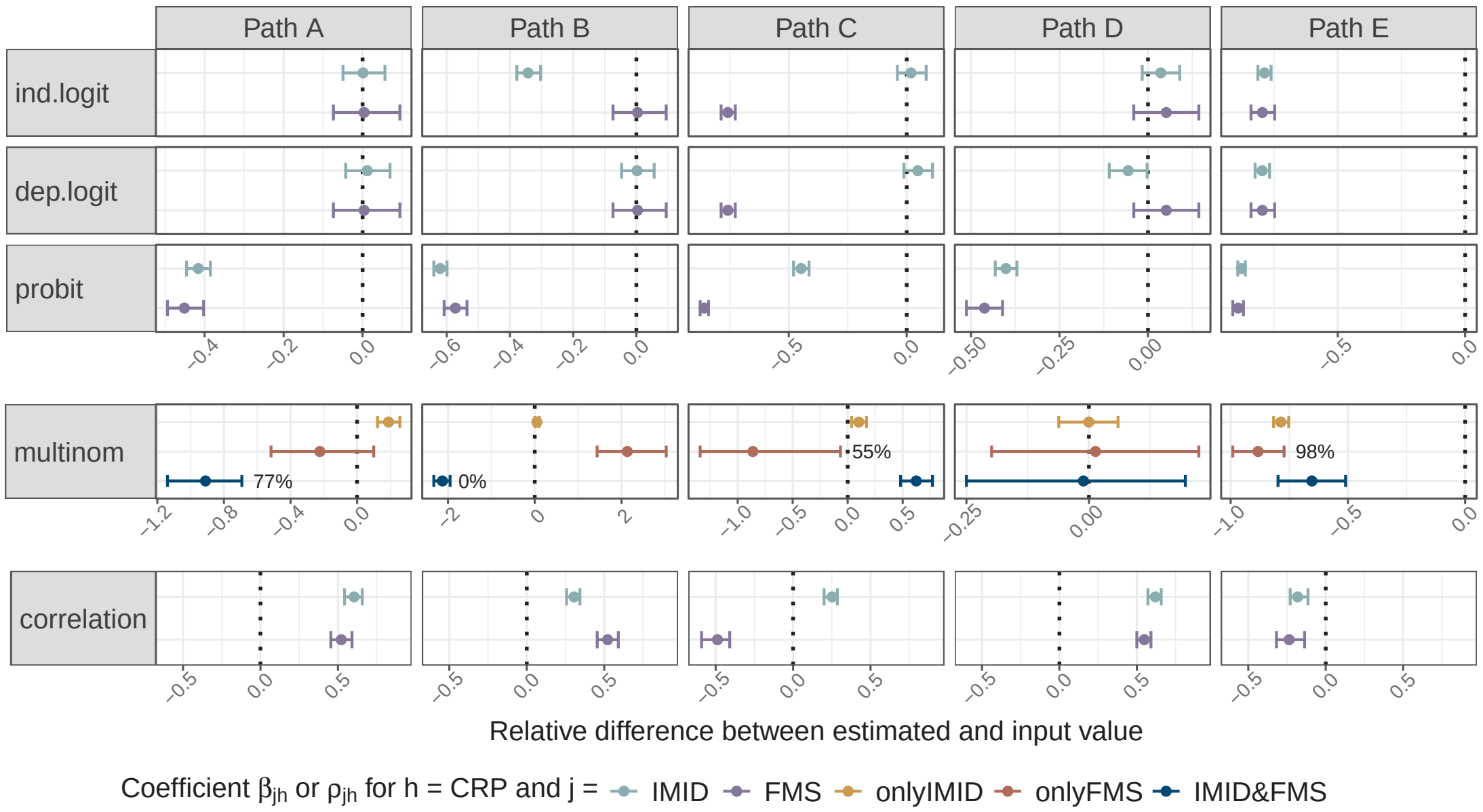

Figure A2: Simulation results as in Figure 2 in the main manuscript, this time for covariate $h = \texttt{CRP}$.

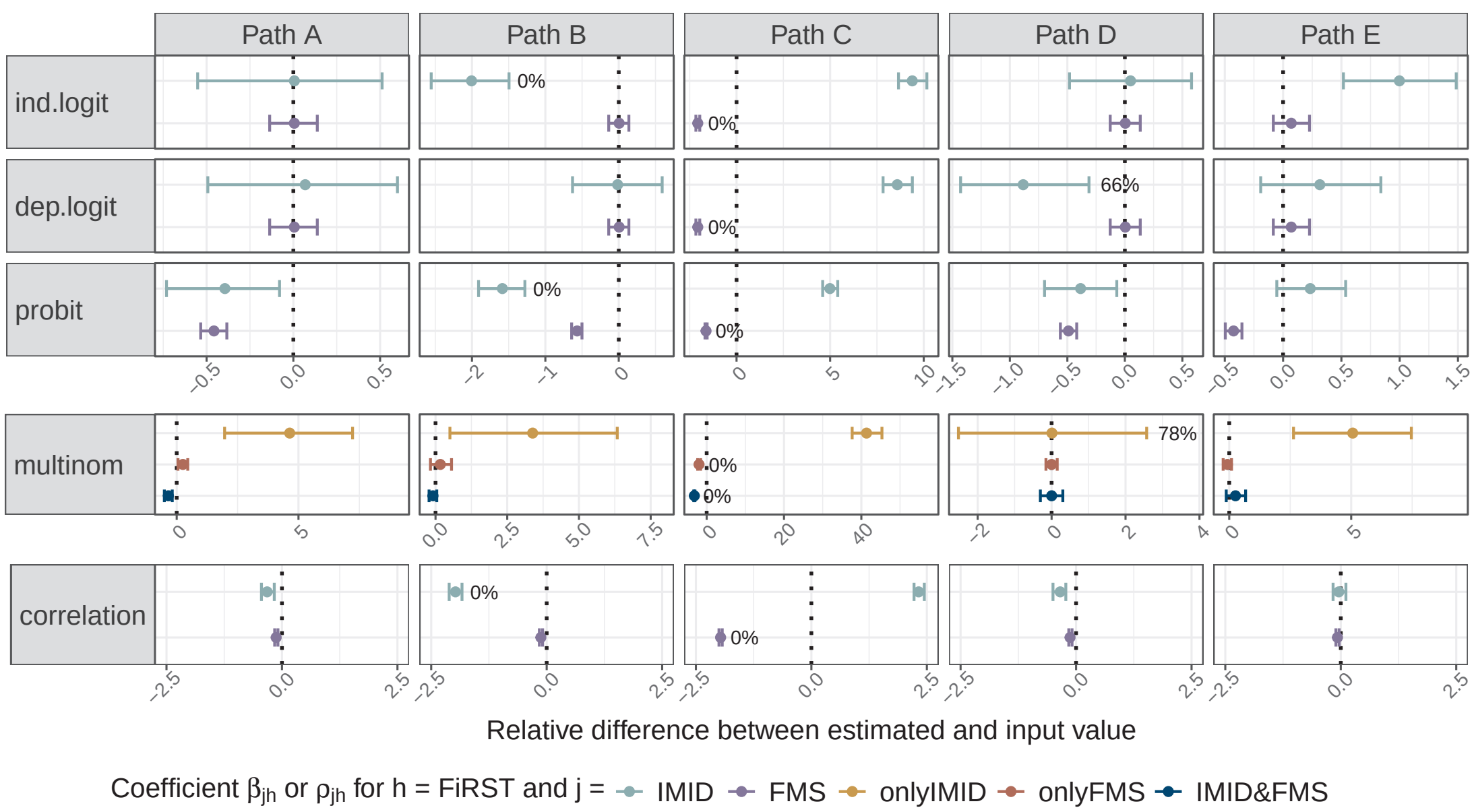

Figure A3: Simulation results as in Figure 2 in the main manuscript, this time for covariate $h = \texttt{FiRST}$.

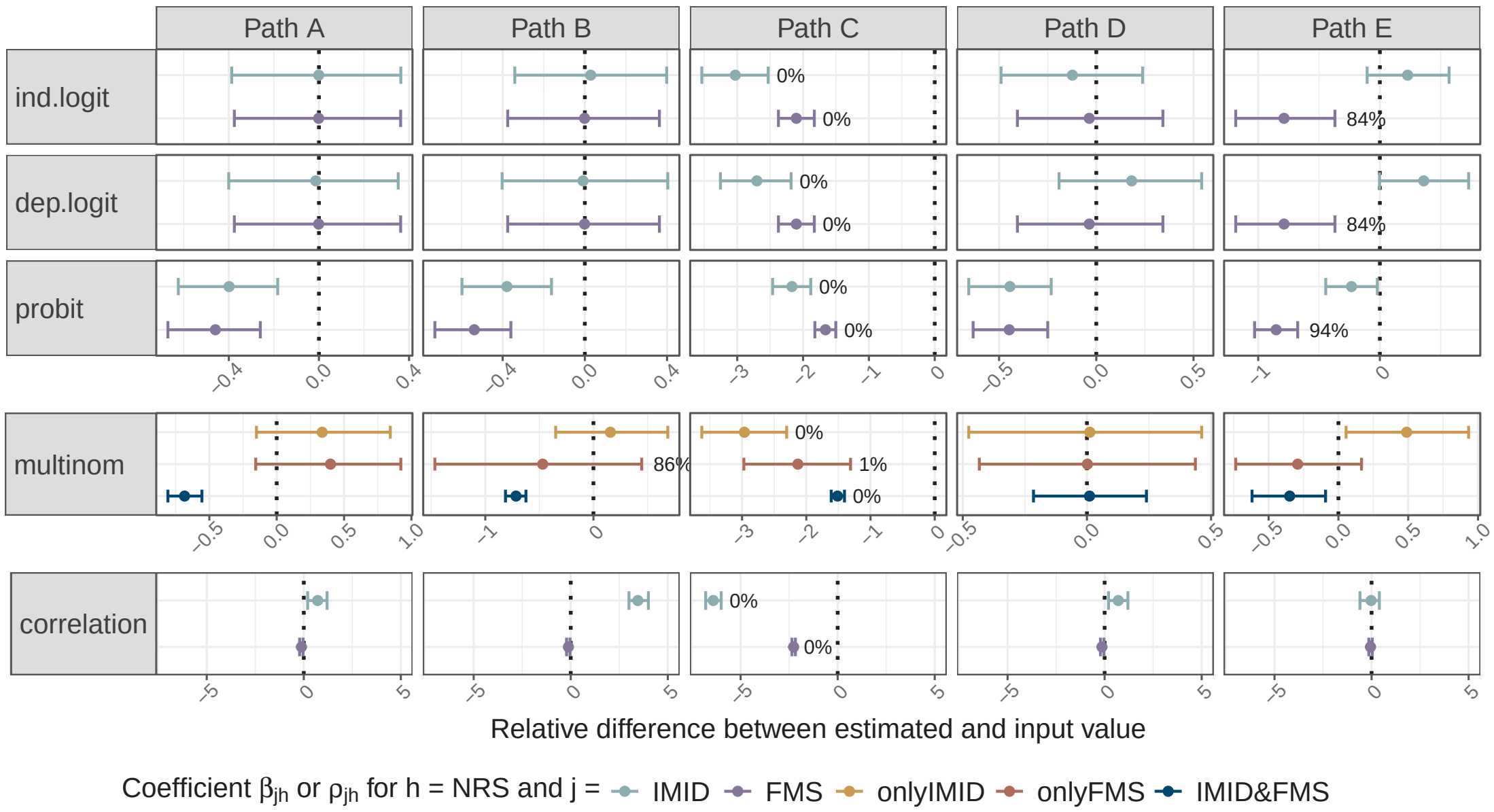

Figure A4: Simulation results as in Figure 2 in the main manuscript, this time for covariate $h =$ `NRS`.

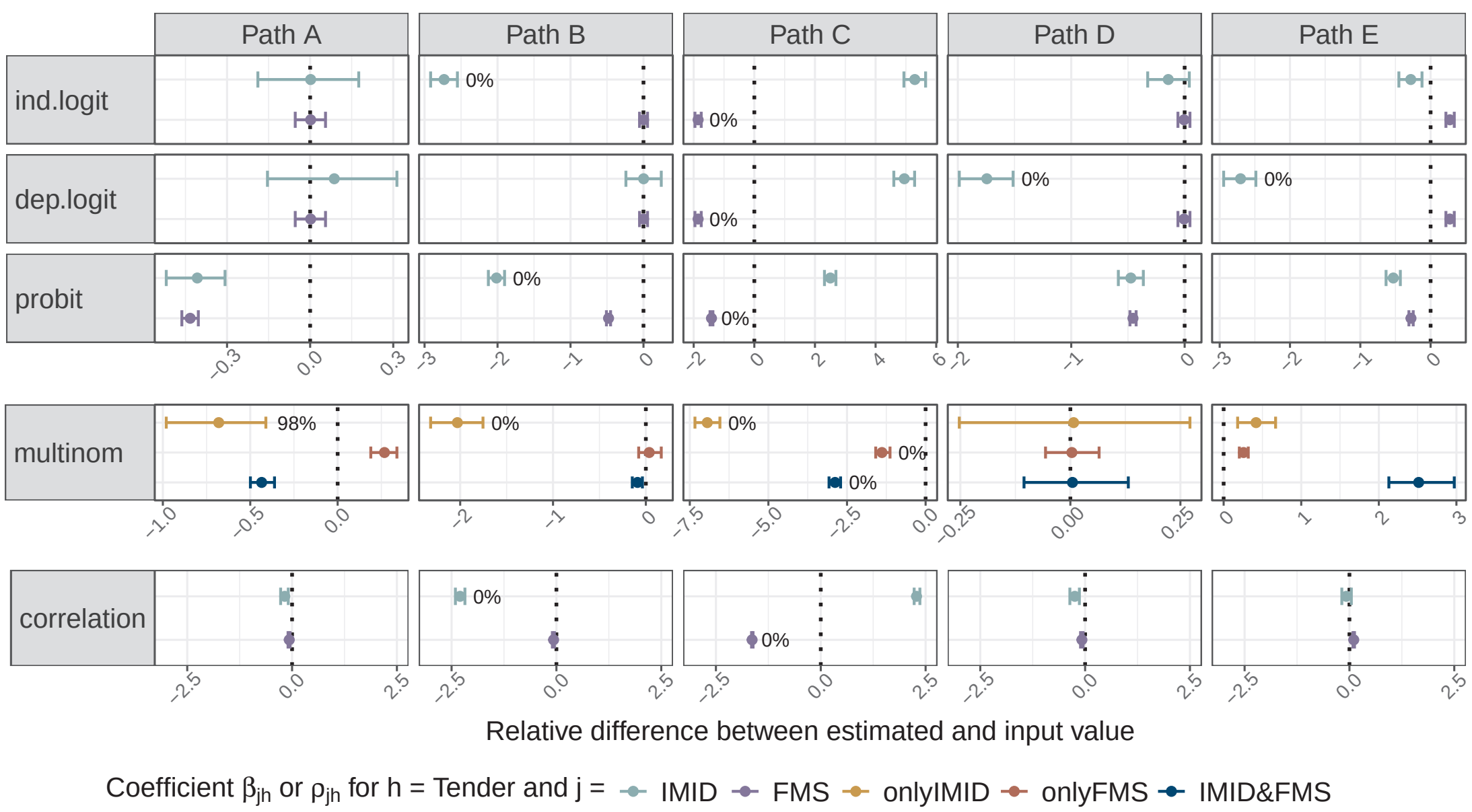

Figure A5: Simulation results as in Figure 2 in the main manuscript, this time for covariate $h =$ `Tender`.

			h = Age		h = Sex		h = CRP		h = FiRST		h = NRS		h = Tender	
**Disease** j	**Path**	**Estimation**	**Mean**	**Diff**	**Mean**	**Diff**	**Mean**	**Diff**	**Mean**	**Diff**	**Mean**	**Diff**	**Mean**	**Diff**
IMID	A	ind.logit	0.17	0.00	0.10	0.00	2.32	0.00	-0.10	0.00	0.14	0.00	-0.31	0.00
IMID	B	dep.logit	0.17	0.00	0.10	0.00	2.32	0.01	-0.10	0.00	0.14	0.00	-0.31	0.00
IMID	C	probit	0.31	0.14	0.94	0.85	1.28	-1.04	-0.62	-0.51	-0.16	-0.30	-1.08	-0.77
FMS	A	ind.logit	-0.45	0.00	-0.87	0.00	-2.05	-0.01	0.64	0.00	0.21	0.00	1.40	0.00
FMS	B	dep.logit	-0.45	0.00	-0.87	0.00	-2.05	-0.01	0.64	0.00	0.21	0.00	1.40	0.00
FMS	C	probit	0.11	0.55	0.63	1.50	-0.29	1.75	-0.40	-1.03	-0.14	-0.35	-0.58	-1.97
onlyIMID	D	multinom	0.15	0.00	-0.04	0.00	2.18	0.00	-0.02	0.00	0.12	0.00	0.30	0.00
onlyFMS	D	multinom	-0.34	0.00	-1.04	-0.01	-1.19	-0.02	0.62	0.00	0.20	0.00	1.57	0.01
IMID&FMS	D	multinom	-0.60	0.00	-0.13	-0.01	1.07	-0.01	0.67	0.00	0.86	0.01	1.45	0.01
IMID	E	correlation	0.20	0.00	0.27	0.00	0.29	-0.06	-0.17	0.01	0.05	0.00	-0.20	0.02
FMS	E	correlation	-0.18	0.00	-0.52	0.00	-0.17	0.05	0.53	-0.04	0.26	-0.02	0.92	0.09

Table A4: Estimated value of the regression coefficient β_{jh} or correlation coefficient ρ_{jh}: average values ('Mean') and their difference to the input value ('Diff') across 1,000 generated datasets. Each row stands for a combination of data generation path and a way to estimate β_{jh} or ρ_{jh}, respectively (see also Figures 2 and 3 in the main manuscript). Columns represent the six covariates h. The target disease j covers IMID, FMS, onlyIMID, onlyFMS and IMID&FMS.

	p_{IMID}		p_{FMS}		p_{onlyIMID}		p_{onlyFMS}		$p_{\text{IMID\&FMS}}$		$V_{\text{IMID,FMS}}$	
	(target: 0.52)		(target: 0.21)		(target: 0.49)		(target: 0.19)		(target: 0.03)		(target: 0.42)	
**Path**	**Mean**	**Diff**	**Mean**	**Diff**	**Mean**	**Diff**	**Mean**	**Diff**	**Mean**	**Diff**	**Mean**	**Diff**
A	0.52	0	0.22	0	0.45	-0.04	0.15	-0.04	0.07	0.04	0.22	-0.20
B	0.52	0	0.22	0	0.32	-0.17	0.02	-0.17	0.20	0.17	0.42	0.00
C	0.52	0	0.22	0	0.32	-0.17	0.02	-0.17	0.20	0.17	0.42	0.00
D	0.52	0	0.22	0	0.49	0.00	0.19	0.00	0.03	0.00	0.42	0.00
E	0.52	0	0.21	0	0.49	0.00	0.18	-0.01	0.03	0.00	0.41	-0.01

Table A5: Empirical disease (combination) prevalence and association in simulated data: average values ('Mean') and their difference ('Diff') to the target value ('target') across 1,000 generated datasets. Each row stands for a data generation path; columns represent the prevalence of diseases or disease combinations (IMID, FMS, onlyIMID, onlyFMS and IMID&FMS) and Cramer's V between IMID and FMS.

References

[1] Sauerbrei W, Blettner M. Interpreting results in 2 x 2 tables: part 9 of a series on evaluation of scientific publications. Deutsches Arzteblatt international. 2009;106(48):795–800. https://doi.org/10.3238/arztebl.2009.0795.

[2] Agresti A. Analysis of Ordinal Categorical Data. 2nd ed. Wiley Series in Probability and Statistics. Hoboken, N.J: Wiley; 2010.

[3] Pearson, Karl. VII. Mathematical contributions to the theory of evolution.—III. Regression, heredity, and panmixia. Philosophical Transactions of the Royal Society of London Series A, Containing Papers of a Mathematical or Physical Character. 1896;187:253–318. https://doi.org/10.1098/rsta.1896.0007.

[4] Drasgow F. Polychoric and Polyserial Correlations. In: Kotz S, Read CB, Balakrishnan N, Vidakovic B, editors. Encyclopedia of Statistical Sciences. Wiley; 2004. .

[5] Fox J.: polycor: Polychoric and Polyserial Correlations. R package version 0.8-1. Available from: https://CRAN.R-project.org/package=polycor.
